# Biological Analysis of Gene Expression and Clinical Variables Suggest FZD1 as a Novel Biomarker for Patients with Kashin-Beck Disease, an Endemic Osteoarthritis in China

**DOI:** 10.1155/2019/3736198

**Published:** 2019-01-03

**Authors:** Xi Wang, Yujie Ning, Pan Zhang, Lei Yang, Cheng Li, Rong Zhou, Xiong Guo

**Affiliations:** ^1^School of Public Health, Xi'an Jiaotong University Health Science Center, Key Laboratory of Trace Elements and Endemic Diseases, National Health and Family Planning Commission, Xi'an 710061, China; ^2^Xi'an Jiaotong University Global Health Institute, Xi'an 710061, China; ^3^School of Nursing, Health Science Center, Xi'an Jiaotong University, Xi'an 710061, China; ^4^Shaanxi Provincial Institute for Endemic Disease Control, Xi'an 710061, China

## Abstract

Clinical variables contribute to the severity of Kashin-Beck disease (KBD). However, it is unclear if there is a correlation between gene expression and clinical variables. Peripheral blood samples were collected from 100 patients with KBD and 100 healthy controls from KBD-endemic areas to identify differentially expressed genes in KBD. Correlation analysis and multiple logistic regression analysis were performed using gene expression and clinical parameters. Immunohistochemistry (IHC) was used to detect the expression of related proteins in articular cartilage tissues. Thirty-nine differentially expressed genes were identified in patients with KBD. Nine differentially expressed genes were correlated with the metacarpal length/metacarpal breadth index. FZD1 was identified as having statistical significance in establishing the regression model of clinical parameters and gene expression. FZD1 expression levels were remarkably reduced in patients with KBD. Our results indicate that FZD1 could be involved in the pathological process of phalanges tuberositas and brachydactylia and may provide new insight into the pathogenesis of articular cartilage destruction observed in patients with KBD.

## 1. Introduction

Kashin-Beck disease (KBD) is a result of lesions in articular cartilage and epiphyseal plate cartilage from necrosis in deep zone chondrocytes, as well as excessive apoptosis and dedifferentiation in chondrocytes, and an important cause of disability in northwestern China [1–3]. According to the clinical features of the disease, including tuberosity of the fingers and brachydactylia, deformed and enlarged joints, and limited motion of the joints, patients with KBD are classified as different grades (I, II, and III) [4, 5]. Although examinations of lesions in articular cartilage and epiphyseal plate cartilage have provided insights into the cellular and molecular mechanisms involved in the necrosis, excessive apoptosis, and dedifferentiation, further exploration is needed to improve the relationship between clinical variables and gene expression in the pathogenesis of KBD.

Some clinical parameters are connected with disease severity in patients with KBD. Patients with limitation of motion, arthralgia, and deformity of the limbs are considered to have advanced KBD [6]. It is possible that the clinical features that represent the KBD grade follow gene expression patterns. Modern molecular medicine has been performed in an exploration to identify genes expressed in the pathologic tissues of KBD. Although research results on pathogenesis in KBD are limited, there are plenty of experimental data in the studies on mechanisms involved in gene expression [7, 8].

However, there are few research studies focused on the relationship between gene expression pattern and clinical parameters in KBD. In this study, we have studied the association between clinical parameters and the gene expression signatures of patients with KBD to test the hypothesis that gene expression is related to patients' clinical parameters. If clinical parameters are significant determinants of gene expression alterations in KBD, these results could provide a molecular insight into understanding the pathogenesis of cartilage injuries and the subsequent development of KBD.

## 2. Materials and Methods

The study was approved by the Ethical Committee of Xi'an Jiaotong University. All participants gave their informed consent by signing a document that had been carefully reviewed by the Ethical Committee of Xi'an Jiaotong University.

### 2.1. Patients and Tissue Samples

Random selection was used for enrolling subjects from Yong-shou and Lin-you counties in Shaanxi Province, two endemic areas of KBD with prevalence rates of 20.4% and 10.5%, respectively. The national diagnostic criteria for KBD in China (WS/T207-2010) was used to diagnose the patients. Healthy controls without clinical symptoms of KBD were collected and matched with KBD patients by gender and age. Subjects with other types of osteoarthropathy and other chronic diseases, such as cardiovascular disease, diabetes, and hypertension, were excluded. As mentioned above, 100 KBD patients (50 degree I and 50 degree II) and 100 healthy controls were selected for this study. The average age of patients was 57.9 years, ranging from 43 to 79 years, and the average age of the controls was 53.4 years, ranging from 40 to 77 years. Both patients and controls consisted of 44 males and 56 females. Peripheral blood was collected from each subject included in this study using heparinized Vacutainer® tubes (Becton Dickenson, San Jose, CA, USA). The Hemavet 950 (Drew Scientific, Oxford, CT, USA) was used for determining leukocyte cell numbers. The peripheral blood was centrifuged at 1500 × g for 20 min to separate peripheral blood mononuclear cells (PBMCs). The cell pellets were resuspended in Hanks' balanced salt solution (Gibco BRL/Invitrogen, Carlsbad, CA, USA). The cell suspensions were layered over 5 mL of lympholyte-H (Cedarlane Labs, Hornby, BC, Canada) in a 15 mL Falcon tube and were centrifuged at 1500 × g for 40 min. The cells were stored in RNAlater® (Ambion Inc., Austin, TX, USA) until RNA isolation after rinsing twice with cold Hanks' balanced salt solution.

According to the inclusion and exclusion criteria of the sample selection described above, the articular cartilage specimens were collected from five patients with KBD and five healthy controls (Table 1). The donors of the adult KBD samples were from the KBD-endemic area of Yong-shou County. Control sample donors were also from the KBD-endemic areas. The articular cartilage samples of the KBD patients and the healthy controls were collected from individuals who had undergone an arthroplasty in the knee or who had suffered an amputation due to an accident.

All of the articular cartilage samples were obtained within 6 h after surgery or patient death. After removing the skin and muscles from the fingers, we opened the joint capsules carefully and harvested articular cartilage. Chondrocytes were isolated as described previously [9, 10].

### 2.2. Clinical Parameter Questionnaire

A KBD questionnaire with a series of clinical variables and general information was completed by all KBD subjects in this study. The clinical variables used for this study included phalanges tuberositas, brachydactylia, the deformity of the elbow and knee, and dyskinesia in joints of the wrist, elbow, shoulder, knee, and ankle. General information included age, gender, BMI, metacarpal length, and metacarpal breadth. The Manouvrier's Skelic Index, metacarpal length/metacarpal breadth index, and metacarpal length/height index were calculated based on the following equations:

(1) $$\begin{array}{c} Manouvrier\text{'}s\;Skelic\;Index=\frac{leg\;length\times \left(height-sitting\;height\right)}{sitting\;height}\times 100,\\ Metacarpal\;length/metacarpal\;breadth\;index=\frac{metacarpal\;length}{metacarpal\;breadth}\times 100,\\ Metacarpal\;length/height\;index=\frac{metacarpal\;length}{height}\times 100. \end{array}$$

### 2.3. Selection of the Candidate Genes

In this study, we reviewed two databases of previously published microarray analyses [7, 11] and selected 169 genes including 11 housekeeping genes as target genes to be measured in a custom-made microarray. From the previously published studies, genes with more than 2- or less than 0.5-fold change in gene expression level between KBD and healthy persons, with a *P* value less than 0.05 and biologic function related to cartilage, were selected. Ultimately, the 158 genes identified by application of the selection criteria above were selected as target genes.

### 2.4. Microarray Hybridization

Total RNA was isolated from the PBMCs using TRIzol® reagent (Life Technologies Inc., Carlsbad, CA, USA) following the manufacturer's recommended protocol. A high-resolution electrophoresis system (Agilent 2100 Bioanalyzer, Agilent Technologies, Palo Alto, CA, USA) was used for determining the quality and integrity of the extracted total RNA. The total RNA isolated from all patients with KBD and healthy controls were transcribed into complementary DNA (cDNA) and then were reverse-transcribed into cRNA and labelled with CyDye using the Amino Allyl Message Amp™ and RNA Amplification Kit (Ambion) according to the manufacturer's instructions. The custom-made primer array contained 169 oligonucleotide probes representing the selected 169 human genes (manufactured by the National Engineering Research Center for Miniaturized Detection System in Xi'an, China). Microarray hybridization was performed following the recommended protocol [12].

### 2.5. Gene Expression Analysis

Expression ratios were calculated for each gene with more than 2-fold change or less than 0.5-fold change that was regarded as statistically significant and considered a differentially expressed gene. *P* values were calculated using the *P* value log ratio algorithm:

(2) $$\begin{array}{c} P=1-Erf\left(\frac{\left|xdev\right|}{\sqrt{2}}\right)=Erfc\left(\frac{\left|xdev\right|}{\sqrt{2}}\right), \end{array}$$

in which Erf(*x*) is calculated as

(3) $$\begin{array}{c} Erf\left(x\right)=\frac{2}{\sqrt{Pi}}\int_{0}^{x}e^{-t^{2}}dt. \end{array}$$

Erf(*x*) represents twice the integral of the Gaussian distribution, with a mean value of 0 and variance of 0.5, and xdev is the deviation of the log ratio from 0. This calculation gives the statistical significance of the log ratio for each feature (i.e., transcript levels) between the red and green channels. Only *P* values less than 0.05 were regarded as significant.

### 2.6. Data Mining and Statistical Analysis

Statistics for comparison and correlation were analysed by SPSS18.0. Differences in categorical variables were evaluated with by Pearson's Chi-square test. Differences of means were determined by one-way analysis of variance (ANOVA) for multiple comparisons. The *t*-test was applied to determine the difference between two groups. A normality test was first applied in continuous variables before any deeper comparison analyses. Nonparametric methods (e.g., Mann-Whitney *U* and Kruskal Wallis tests for pairwise comparisons) were applied when the data were not normally distributed. Correlation analysis was used between gene expression and indexes of clinical parameters, such as the Manouvrier's Skelic Index, the metacarpal length/metacarpal breadth index, and the metacarpal length/height index. Univariate and multiple logistic regression analyses were used to identify the clinical sign-specific genes.

### 2.7. Immunohistochemical Localization Verification in Cartilage Tissues from Patients with KBD

Cartilage tissues were fixed with 4% paraformaldehyde for 24 hours after removal of the tissue and decalcified in 10% ethylenediaminetetraacetic acid (EDTA). The samples were dehydrated in a series of alcohol, cleared in xylene, and embedded in paraffin wax. Paraffin sections were cut into 5 *μ*m sections, mounted on slides, and stored at room temperature until used for staining. The paraffin-embedded sections were deparaffinized with xylene and then rehydrated in decreasing graded ethanol. Endogenous peroxidase activity was blocked by 3% hydrogen peroxide for 10 min at room temperature, then the sections were washed with PBS and incubated in 10 M urea solution and trypsin at 37°C for 20 min to unmask the antigen. After blocking with 5% goat serum for 20 min at room temperature, anti-FZD1 (1 : 50 dilution), as well as negative control IgG, were applied on the sections, and the samples were incubated overnight at 4°C. After washing with PBS, sections were incubated using the SAP kit (Zhongshan Jinqiao, Guangzhou, China). The substrate 3, 3′-diaminobenzidine (DAB) was added to stain the sections with haematoxylin counterstaining. Finally, the sections were dehydrated and mounted under alcohol-cleaned cover slips. The percentage of positive cells in different zones of cartilage was calculated using the Kuettner standard [13] for defining the zones. The localizations of FZD1 in each cartilage zone were assessed systematically by the rate of positively stained cells calculated by the equation below:

(4) $$\begin{array}{c} \text{The }percentage\text{ of }positive\;cells=\frac{positively\;stained\;cells}{\left(positively\;stained\;cells\right)+\left(negatively\;stained\;cells\right)}\times 100\%. \end{array}$$

## 3. Results and Discussion

After checking whether the questionnaires were consistent with the inclusion and exclusion criterion by two well-trained research assistants, eighty questionnaires of patients with KBD were ultimately included in this analysis.

### 3.1. Univariate Analysis in Clinical Parameters and General Information

Univariate analysis was applied in clinical parameters and general information. There were significant differences between KBD patients with grade I and II disease in four items among nine clinical parameters (brachydactylia, *P* = 0.0001; deformity of the knee, *P* = 0.004; and dyskinesia in joints of the elbow and knee, *P* = 0.019; *P* = 0.0001) (Table 2), and only four items among fourteen general information items were significantly different between KBD patients with grade I and II disease (age, *P* = 0.034; metacarpal length, *P* < 0.001; blurred vision, *P* = 0.03; and number of family members with KBD, *P* = 0.003) (Tables 2 and 3).

### 3.2. Differentially Expressed Genes

39 differentially expressed genes, including 21 downregulated and 18 upregulated genes, were identified in the PBMCs of patients with KBD (Table 4). There were 12 differentially expressed genes in KBD grade I patients, and 92 differentially expressed genes were identified in KBD grade II patients. These genes belong to different functional pathways, including metabolism, transcription factors, cytokine factors, and signal transduction.

### 3.3. Correlation Analysis and Univariate Analysis in Gene Expression and Clinical Parameter Indexes

The correlation analysis was performed to identify the relationship between differentially expressed genes and clinical parameter indexes. We found that only nine genes among the 39 differentially expressed genes were correlated with the metacarpal length/metacarpal breadth index (Table 5). By using Pearson's chi-squared test, the differential expression rate of all nine genes was identified as statistically significant between KBD grades I and II (Table 6), six genes were identified as statistically significant in phalanges tuberositas (Table 7), and eight genes were identified as statistically significant in brachydactylia (Table 8).

### 3.4. Multiple Logistic Regression Analysis of Clinical Symptoms (Phalanges Tuberositas and Brachydactylia) and Differentially Expressed Genes

We used the six and eight genes that were identified as having a statistically significant correlation with the clinical symptom by univariate regression analysis and multiple logistic regression analysis. Clinical signs (phalanges tuberositas and brachydactylia) were considered as dependent variables, and differentially expressed genes were considered as independent variables in multiple logistic regression analysis. Finally, we found that FZD1 and TNFSF11 genes were identified as statistically significant in establishing the regression model of phalanges tuberositas and gene expression (Table 9). The FZD1 gene was identified as a statistically significant factor in establishing the regression model of brachydactylia and gene expression (Table 10).

### 3.5. The Expression of FZD1 in Cartilage Tissue from Patients with KBD

**Figure 1:**
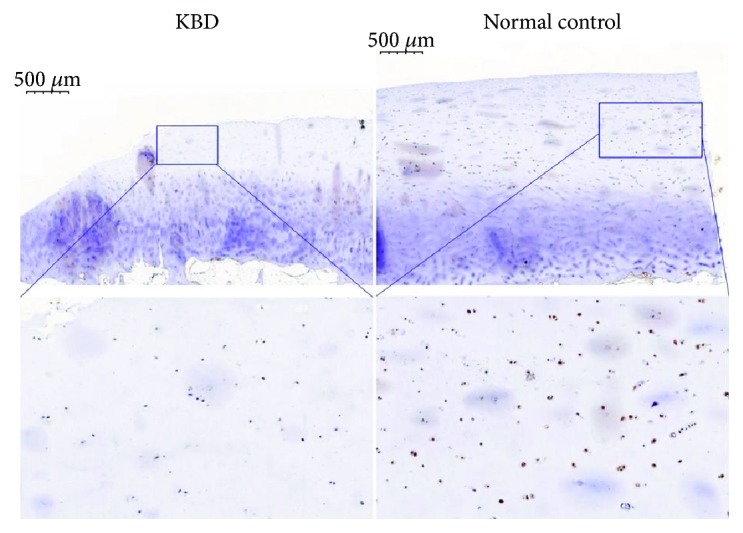
FZD1 immunostaining in the articular cartilage from the KBD and the normal controls (positive staining is yellow).

To evaluate the expression level of FZD1 in patients with KBD, FZD1 in articular cartilage tissues was detected using immunohistochemistry (IHC). In cartilage, positive staining for FZD1 was found mainly in the cytomembrane and cytoplasm of the superficial and middle zones of articular cartilage from the adult control group. FZD1-positive staining was lower in the superficial and middle zones in the adult KBD samples than in the adult control samples (Figure 1).

This study first investigated the comprehensive gene expression of patients with KBD in relation to the patients' clinical variables. The most important finding is that the expression levels of FZD1 and TNFSF11 were statistically significant in establishing the regression model of phalanges tuberositas, brachydactylia, and gene expression. Our study suggests that the molecular and biological effect of KBD could be linked with KBD clinical variables.

At present, the diagnosis and determination of KBD grades are primarily dependent on symptoms and changes observed by X-ray, but it is only effective for diagnosing the advanced cases of KBD with grades II and III. Thus, it is extremely difficult to correctly diagnose the early stages of KBD with grades I and II, leading to inevitable exacerbation of symptoms [5, 14]. In this study, we found that brachydactylia, deformity of the knee, and dyskinesia in joints of the elbow and knee were significantly different between KBD patients with grades I and II, which could provide the new indicators for diagnosis of KBD patients with grades I and II.

The Manouvrier's Skelic Index, metacarpal length/metacarpal breadth index, and metacarpal length/height index were used for reflecting the severity of KBD. The patients with KBD included in this study mainly consisted of grade I and II patients, whose lesions mainly occurred on their hands and joints. Therefore, gene correlation analysis showed that TNFSF11, GDF5, CTSC, and FZD1 were strongly and significantly associated with metacarpal length/metacarpal breadth index regardless of the Manouvrier's Skelic Index and metacarpal length/height index.

Differential expression rates of a set of genes (ATR, CSGALNACT, CTSC, COL1A1, FZD1, GDF5, SLC14A1, SSBP1, and TNFSF11) were associated with KBD grades I and II; these genes tended towards abnormal expression in grades II, and all of these genes were differentially expressed in patients with phalanges tuberositas and brachydactylia except CSGALNACT, COL1A1, and SSBP1. Some of the genes above have also been reported to be differentially expressed in chondrocytes from patients with KBD [1, 5, 10]. This observation is consistent with our analysis and suggests that the involvement of these genes in pathophysiological changes is associated with clinical variables in KBD.

Ageing and joint disease are characterized by disruption of the equilibrium within cartilage tissue, in which the synthesis of new matrix components is more than the loss of collagens and proteoglycans [15–17]. This imbalance between anabolic and catabolic processes may result in progressive cartilage degeneration. The Wnt signalling pathway, composed of several important components, such as frizzled, a cell membrane receptor [18–20], plays an important role in embryonic development, skeletal development, and the maintenance of cartilage homeostasis [21–24]. Cartilage homeostasis in healthy joints is maintained by the balance of synthesis and degradation of extracellular matrix (ECM). Cartilage undergoes destruction when this balance is lost. In this study, we found that FZD1 (a member of the frizzled family) was downregulated in the peripheral blood of KBD patients and the expression level of FZD1 was associated with phalanges tuberositas and brachydactylia, two clinical symptoms of KBD analysed by multiple logistic regression analysis in the current study. Additionally, IHC was performed to evaluate the expression of these genes in articular cartilage from adult and child patients with KBD. From the results of IHC, expression levels of FZD1 were significantly reduced in patients with KBD compared with normal controls. It suggested that the abnormal expression of FZD1 in the articular cartilage of patients with KBD results in disequilibrium of the anabolism and catabolism of ECM by abnormally activating the Wnt signalling pathway. This disequilibrium in ECM involved in the abnormal expression of FZD1 could be one of the reasons for the process of pathological change in phalanges tuberositas.

Apoptotic chondrocytes are increased in the middle zone of articular cartilage in adult KBD patients in comparison to healthy controls, with the exception of multiple focal chondronecrosis [6, 25]. It suggested that the signal for apoptosis has already occurred in the middle zone of articular cartilage in KBD children. In previous studies, Bcl-2, Bax, and Fas were expressed in enhanced amounts in the upper zone and middle zone, and iNos expression was more abundant in the entire articular cartilage [6]. The TNFSF gene encodes a member of the tumor necrosis factor (TNF) cytokine family which is a ligand for osteoprotegerin and functions as a key factor for osteoclast differentiation and activation. This protein was shown to activate antiapoptotic kinase AKT/PKB through a signalling complex involving SRC kinase and tumor necrosis factor receptor-associated factor (TRAF) 6, which indicated this protein may have a role in the regulation of cell apoptosis [26, 27]. In this study, we found that TNFSF11 was upregulated in the peripheral blood of KBD patients and the expression level of TNFSF11 was associated with phalanges tuberositas in multiple logistic regression analysis. These results suggest that the abnormal expression of relevant chondrocyte molecules triggered apoptosis in KBD cartilage. Abnormal chondrocyte apoptosis can not only affect the development of bone and cartilage but also lead to phalanges tuberositas.

## 4. Conclusions

In summary, our study first investigated the correlation between gene expression and clinical parameter indexes in KBD. The results from multiple logistic regression analysis and immunohistochemistry indicate that FZD1 could be involved in the process of pathological change in phalanges tuberositas/brachydactylia and may provide further new information contributing to explanations for the articular cartilage destruction and pathology observed in patients with KBD.

## Figures and Tables

**Table 1:** Cartilage tissue and chondrocyte from the patients with KBD and control. Cartilage tissue collection and chondrocyte culture.

Sample pair	KBD		Normal	
	Age (years)	Gender	Age (years)	Gender
1	61	Female	60	Female
2	62	Female	63	Female
3	59	Male	58	Male
4	56	Female	52	Female
5	54	Male	55	Male
Mean	58.4		57.6	

**Table 2:** Univariate analysis of categorical variables in clinical parameters and general information between KBD grades I and II.

Variables	Grade I		Grade II		*χ* ^2^	*P*	OR
	+	−	+	−			
Phalanges tuberositas	23	7	47	3	3.688	0.055	4.768 (1.13 ± 20.15)
Brachydactylia	3	27	49	1	63.82	0.0001	441.0 (43.7 ± 4449)
Deformity of elbow	23	7	47	3	3.688	0.055	4.768 (1.13 ± 20.15)
Deformity of knee	11	19	35	15	8.525	0.004	4.030 (1.54 ± 10.50)
Dyskinesia in joints of wrist	22	8	46	4	3.765	0.052	4.182 (1.13 ± 15.39)
Dyskinesia in joints of elbow	24	6	49	1	5.521	0.019	12.25 (1.39 ± 107.5)
Dyskinesia in joints of shoulder	1	29	11	39	3.765	0.052	8.179 (0.99 ± 66.98)
Dyskinesia in joints of knee	11	19	40	10	15.23	0.0001	6.909 (2.50 ± 19.07)
Dyskinesia in joints of ankle	26	4	48	2	1.201	0.273	3.692 (0.63 ± 21.53)
Fracture	2	28	6	44	0.148	0.700	1.909 (0.36 ± 10.13)
Blurred vision	18	12	41	9	4.688	0.030	3.037 (1.08 ± 8.479)
Hearing disorder	13	17	19	31	0.222	0.637	0.081 (0.32 ± 2.012)
Diarrhea	7	23	4	46	3.717	0.054	0.286 (0.07 ± 1.077)
Astriction	2	28	0	50	—	0.138	0.933 (0.84 ± 1.027)
Senile wart	7	23	10	40	0.124	0.724	0.821 (0.27 ± 2.452)
Decayed tooth	28	2	39	11	2.210	0.137	0.253 (0.05 ± 1.233)
Smoke	8	22	12	38	0.071	0.790	0.868 (0.31 ± 2.450)
Alcohol consumption	0	30	4	46	—	0.291	1.087 (1.01 ± 1.180)
Patients with KBD in other family members	10	20	34	16	9.104	0.003	4.250 (1.62 ± 11.14)

OR: odds ratios.

**Table 3:** Univariate analysis (Mann-Whitney *U* test) of continuous variables in clinical parameters and general information between patients with KBD grades I and II.

Variables	Total	Grade I	Grade II	*Z*	*P*
		$\bar{X}+S$	$\bar{X}+S$		
Age	57.0 ± 7.3	55.3 ± 9.2	58.0 ± 5.7	−2.120	0.034
BMI index	21.7 ± 2.6	21.7 ± 2.1	21.5 ± 2.8	−0.924	0.355
Metacarpal length	16.2 ± 1.2	16.7 ± 1.2	15.8 ± 1.1	−3.496	0.000
Metacarpal breadth	8.14 ± 0.5	8.01 ± 0.6	8.22 ± 0.5	−1.489	0.137

**Table 4:** List of 39 differentially expressed genes in KBD patients.

Gene name	Symbol	Public ID	Fold change^a^
*Downregulated genes*			
ATP-binding cassette, subfamily C, member 13, pseudogene	ABCC13	NR_003087	0.42 ± 0.03
ABI family, member 3 (NESH) binding protein	ABI3BP	NM_015429	0.33 ± 0.03
Acyl-CoA synthetase long-chain family member 6	ACSL6	NM_001009185	0.31 ± 0.01
Anaphase promoting complex subunit 1	ANAPC1	XM_006712690	0.33 ± 0.02
Branched chain amino-acid transaminase 1, cytosolic	BCAT1	NM_001178091	0.43 ± 0.04
Chromosome 1 open reading frame 64	C1orf64	NM_178840	0.43 ± 0.03
Calcium channel, voltage-dependent, gamma subunit 6	CACNG6	NM_145814	0.39 ± 0.02
Chondroitin sulfate N-acetylgalactosaminyltransferase 1	CSGALNACT1	NM_001130518	0.45 ± 0.03
Cathepsin C	CTSC	NM_001114173	0.39 ± 0.02
Cytochrome b5 reductase 3	CYB5R3	NM_000398	0.48 ± 0.04
Dystrophin, muscular dystrophy	DMD	NM_007868	0.37 ± 0.02
Enhancer of rudimentary homolog (Drosophila)	ERH	NM_004450	0.38 ± 0.02
F11 receptor	F11R	NM_016946	0.46 ± 0.02
Fibrillin 1	FKBP9	NM_007270	0.49 ± 0.06
Frizzled family receptor 1	FZD1	NM_003505	0.47 ± 0.03
Growth differentiation factor 5	GDF5	NM_000557	0.44 ± 0.03
Glutaredoxin 5	GLRX5	NM_016417	0.46 ± 0.04
Hemoglobin, alpha 2	HBA2	NM_000517	0.49 ± 0.03
Mitochondrial carrier triple repeat 3 pseudogene	MCART3P	NR_026540	0.43 ± 0.03
STE20-related kinase adaptor beta	STRADB	NM_001206864	0.43 ± 0.03
Zic family member 5	ZIC5	NM_033132	0.36 ± 0.02
*Upregulated genes*			
Aquaporin 1	ATR	NM_001184	2.84 ± 0.27
Baculoviral IAP repeat-containing 3	BIRC3	NM_001165	4.26 ± 0.35
Collagen, type I, alpha 1	COL1A1	NM_000088	2.44 ± 0.44
CD3g molecule, gamma (CD3-TCR complex)	CD3G	NM_000073	3.06 ± 0.25
EYA transcriptional coactivator and phosphatase 4	EYA4	NM_001301012	3.77 ± 0.53
Fem-1 homolog a (*C. elegans*)	FEM1A	NM_018708	2.68 ± 0.49
F-box protein 15	FBXO15	NM_001142958	2.59 ± 0.18
FGFR1 oncogene partner 2	FGFR1OP2	NM_001171887	3.19 ± 0.24
Small subunit (SSU) processed component, homolog	KRR1	NM_007043	2.25 ± 0.19
Optineurin	OPTN	NM_001008211	2.01 ± 0.14
Phosphodiesterase 8B	PDE8B	XM_005248623	2.79 ± 0.16
Required for meiotic nuclear division 5 homolog A	RMND5A	NM_0022780	2.90 ± 0.43
Sialic acid binding Ig-like lectin 8	SIGLEC8	NM_014442	2.49 ± 0.29
Solute carrier family 14 (urea transporter), member 1	SLC14A1	NM_001128588	2.47 ± 0.22
Spermatogenesis associated 22	SPATA22	NM_001170695	2.06 ± 0.21
Single-stranded DNA binding protein 1, mitochondrial	SSBP1	NM_001256510	3.12 ± 0.25
Tumor necrosis factor (ligand) superfamily, member 11	TNFSF11	NM_003701	3.30 ± 0.53
Tetratricopeptide repeat domain 25	TTC25	NM_031421	2.52 ± 0.15

Differential expression genes between the KBD patients vs. controls were assessed using the selection criteria described in the Materials and Methods. According to the fold change value, only those genes showing significant differences (*P* value < 0.05) in expression, screening for the differential expression genes in KBD, are listed. ^a^Fold change, the mean and standard error of the mean (SEM) of the fold change in the expression of each gene.

**Table 5:** Correlation analysis between gene expression and metacarpal length/metacarpal breadth index.

Gene name	*R*	*P*
ATR	0.401	0.001
CSGALNACT	−0.457	0.001
CTSC	−0.504	0.001
COL1A1	−0.424	0.001
FZD1	−0.497	0.001
GDF5	−0.537	0.001
SLC14A1	0.464	0.001
SSBP1	0.270	0.027
TNFSF11	0.564	0.008

**Table 6:** Differential expression rate of nine differentially expressed genes in patients with KBD grades I and II.

Gene	Grade I		Grade II		*χ* ^2^	*P*	OR
	+	−	+	−			
ATR	3	27	33	17	23.76	0.001	17.4 (4.62 ± 65.96)
CSGALNACT	8	22	48	2	42.92	0.001	66.0 (12.93 ± 336.71)
CTSC	8	22	49	1	46.57	0.001	134.7 (15.87 ± 1143.71)
COL1A1	9	21	48	2	39.87	0.001	56.0 (11.13 ± 281.76)
FZD1	9	21	49	1	43.48	0.001	114.3 (13.61 ± 960.45)
GDF5	9	21	49	1	43.48	0.001	114.3 (13.61 ± 960.45)
SLC14A1	1	29	33	17	30.13	0.001	56.2 (7.05 ± 449.51)
SSBP1	9	21	48	2	39.87	0.001	56.0 (11.13 ± 281.76)
TNFSF11	1	29	32	18	28.47	0.001	51.5 (6.47 ± 410.79)

OR: odds ratios.

**Table 7:** Univariate analysis between nine differentially expressed genes and phalanges tuberositas.

Gene		Phalanges tuberositas		*χ* ^2^	*P*	OR
		+	−			
ATR	+	35	1	5.65	0.041	9.0 (1.08 ± 74.86)
	−	35	9			
CSGALNACT	+	18	0	3.31	0.157	—
	−	52	10			
COL1A1	+	54	2	13.61	0.001	13.5 (2.61 ± 70.04)
	−	16	8			
CTSC	+	55	2	14.65	0.001	14.6 (2.81 ± 76.47)
	−	15	8			
FZD1	+	56	2	15.79	0.001	16.0 (3.05 ± 83.85)
	−	14	8			
GDF5	+	56	2	15.79	0.001	16.0 (3.05 ± 83.85)
	−	14	8			
SLC14A1	+	51	3	2.36	0.231	3.3 (0.66 ± 17.00)
	−	19	7			
TNFSF11	+	32	2	11.29	0.003	9.3 (2.13 ± 40.75)
	−	38	8			
SSBP1	+	31	2	2.13	0.144	3.2 (0.62 ± 16.06)
	−	39	8			

OR: odds ratios.

**Table 8:** Univariate analysis between nine differentially expressed genes and brachydactylia.

Gene		Brachydactylia		*χ* ^2^	*P*	OR
		+	−			
ATR	+	33	3	20.46	0.001	14.4 (3.85 ± 54.39)
	−	19	25			
COL1A	+	12	6	0.028	0.866	1.1 (0.36 ± 3.33)
	−	40	22			
CSGALNACT	+	49	7	41.53	0.001	49.0 (11.54 ± 208.03)
	−	3	21			
CTSC	+	50	7	44.98	0.001	75.0 (14.37 ± 391.32)
	−	2	21			
FZD1	+	50	8	41.69	0.001	62.5 (12.19 ± 320.25)
	−	2	20			
GDF5	+	50	8	41.69	0.001	62.5 (12.19 ± 320.25)
	−	2	20			
SLC14A1	+	32	2	22.03	0.001	20.8 (4.44 ± 97.31)
	−	20	26			
SSBP1	+	50	9	38.52	0.001	52.7 (10.43 ± 266.86)
	−	2	19			
TNFSF11	+	31	2	20.67	0.001	19.1 (4.11 ± 89.62)
	−	21	26			

OR: odds ratios.

**Table 9:** Multiple logistic regression analysis in phalanges tuberositas and differentially expressed genes.

Gene	*B*	S.E.	Sig	OR
ATR	−1.41	2.30	0.539	2.916
COL1A1	−0.59	6.99	0.268	0.83
CTSC	1.48	9.83	0.956	2.13
FZD1	3.95	2.11	0.036	11.54
GDF5	3.62	5.23	0.064	0.22
TNFSF11	−3.44	1.94	0.035	12.15

S.E.: standard error; OR: odds ratios.

**Table 10:** Multiple logistic regression analysis in brachydactylia and differentially expressed genes.

Gene	*B*	S.E.	Sig	OR
ATR	0.99	1.05	0.753	0.51
CSGALNACT	0.56	2.93	0.453	0.02
CTSC	1.61	0.91	0.211	3.64
FZD1	2.44	0.991	0.014	11.54
GDF5	−16.03	7.41	0.803	0.22
SLC14A1	0.027	0.68	0.869	0.74
SSBP1	0.99	0.714	0.319	1.88
TNFSF11	0.62	0.99	0.814	0.54

S.E.: standard error; OR: odds ratios.

## Data Availability

The gene expression data used to support the findings of this study can be searched at https://www.ncbi.nlm.nih.gov/geo/query/acc.cgi?acc=GSE59446.
